# Why Has the American Dentist a High Reputation Throughout the Civilized World?

**Published:** 1891-02

**Authors:** C. T. Terry

**Affiliations:** Milan, Italy


### WHY HAS THE AMERICAN DENTIST A HIGH REPUTATION THROUGHOUT THE CIVILIZED WORLD?[1]

BY DR. C. T. TERRY, MILAN, ITALY.

It is undeniable that we have enjoyed this reputation for many years in all foreign countries as well as in our own. Has it been on account—as many suppose—of our superior dental schools?

We must admit that all schools or colleges for instruction in the professions and sciences are, like common schools for the people, necessary, in order to give all a foundation on which to build, that they may be able to gain at least a livelihood in whichever speciality they have chosen, if not a reputation and fortune.

The man of energy and brain keeps on building on this foundation until disease or old age demands a pause; but, how many there are who receive diplomas, who are not as competent five years after receiving them to practise their professions as they were at the time the diplomas were given. Why? Because they were forced to muster a little energy to get these diplomas, which exhausted all the strength they had and all they ever were to have in this direction, compelling them to fall back on their diplomas alone for a reputation.

In the twenty-five years or more that I have been practising abroad, the question has been asked me perhaps hundreds of times, "Why are the Americans so skilful as dentists?" and how many times have I regretted that I could not answer this question to my satisfaction for want of time.

The general impression prevails that our dental colleges have made us what we are. If our schools have really done this, why did we have a reputation in Europe before a single institution existed in our country for teaching dentistry?

Dr. Brewster established the American dentist's reputation abroad before we had a dental college. Why have we now, and why have we had for years, so many competent dentists and excellent operators who never received instruction in a dental college? If the dental schools can invariably produce excellent operators, why are there so many dentists holding diplomas who cannot hold a dental instrument with steadiness? It is evident that it is not

[1] Read at the Union Meeting, Springfield, Mass., October, 1889.

possible for dental schools to make good dentists out of bad material, any more than it is possible for a literary institution to make all its students poets.

The men of our country who have invented ingenious machines and instruments—many of which have won the admiration of the world—have not been, as a rule, educated in schools of technology.

Wherever you go on the continent, you will see the results of their study, hard work, and ingenious brains. It must be admitted by those who are honest and unprejudiced that great executive ability and superior ingenuity are more general traits among the people of the *United States* than among those of any other civilized country.

When a European comes to an American dentist abroad for treatment, he does not expect to find a mountain of learning or a profound philosopher, but a thoroughly practical man, who won't keep him running a month in order to have the cotton changed which has been crowded between his teeth to get space enough to put your finger in before the cavity can be filled, and toothache enough to drive him wild, but a man who will go right to the spot and do execution. The patient knows by every touch and movement that he has got hold of some one who understands his profession, practically at least. Why is it that there exists so much ability of this kind among the people of the United States?

One very good reason is, that we are descended mainly from one of the most practical nations of Europe, and we germinated in a great, free, and new country, where labor was and is respected and encouraged, and where the intelligent class are obliged to help themselves more than in any other country.

Our forefathers were obliged to develop the inventive ability they brought to the new country, in devising labor-saving machines, and their inventions were appreciated, as laborers were scarce. The inventors were respected and their inventions protected by law more than in any other country; therefore, the inventive ability which was sown here has been encouraged until it has developed into a national talent or trait.

Any American who goes abroad cannot help noticing the want of practical ability among the European laborers. The greatest effort appears to be made among them to do things in the most awkward way possible, while our American-born laborers use their brains to ease their muscles.

The peasants and ordinary laborers of Europe make no effort, apparently, to invent labor-saving machinery, and even if they had the ability, I doubt if they would use it for fear of such machines taking the labor from them.

The aristocracy are very conservative about making the acquaintance of or introducing new things; therefore inventive ability has only been developed among the middle or manufacturing class.

Dentistry as a calling has never been respected on the continent; in any case, not as other learned professions are; and an aristocrat, as far as my experience goes, would not think of educating his son in dentistry. The English aristocracy do not recognize their dentist away from his rooms, notwithstanding that they may have been treated most skilfully and faithfully by him. This I have been told by one of our best and most respected dentists in London.

Callings that are not respected cannot expect to attract as much talent as others, though they may be more remunerative.

Our regard in the United States for all occupations gave dentistry a chance to make a fair start; therefore it immediately called to its field men of talent and superior practical ability; and I believe I am correct in stating that the dental profession in this country can boast of being represented by just as much talent as any other profession.

Its members soon saw the importance of establishing dental schools where a solid foundation could be laid, on which to build a monument composed of the skill, inventive ability, and liberal endeavor of the American dentists of the nineteenth century, which will honor them forever.

What course could be more admirable or more quickly insure a rapid advance in dentistry than that which our more talented brothers in the profession in this country have pursued and are still pursuing towards the less gifted members, in imparting as much knowledge of their ways of practising as possible, and in acknowledging that there should be no secrets from them, excepting in so far as lack of ability prevents them from seeing through and adopting the methods which have been so liberally shown?

How different is the course pursued by foreign dentists! In all the years of my practice abroad, I have never received an invitation from a continental dentist to look on while he operated. This may be accounted for partially by the continental patient's dislike to having others present while being treated, also on account of the dentist's fear of displeasing his patient, or perhaps on account of his own modesty, or that his wonderful method of operating might be stolen! The continental mind is exceedingly cautious and conservative, and circumstances are not favorable for a rapid practical advance in our specialty; but more is being done at present than ever before, as dental schools are springing up in different parts of the continent, which will establish a higher average in the profession. I am of the opinion that the European dentist has a better theoretical education than we possess (excepting our very *best* dentists, who stand far above all—at home and abroad—both in theoretical and practical knowledge).

Dentistry is thoroughly a practical profession. When a dentist wishes an operation performed in his own mouth, how little he cares for his dental brother's theoretical ability! He wishes to know above all things that he is a superior operator.

This superior ability to operate has given us our reputation, and I believe it is a great mistake for our colleges to decorate so many of their students with diplomas who have not practical ability. As soon as we begin to undervalue this, we may expect to see the gradual decay of our reputations.

I think there is no doubt but that the treatment and cure of serious diseases, both of the body as well as the teeth, will, in the future, be more mechanical than medical, if it is not so already, and the nation which possesses the greatest amount of mechanical skill, all things being equal, will be able to do most for the ills and misery of mankind.

